# Cytoprotective Effect of* Lygodium venustum* Sw. (Lygodiaceae) against Mercurium Chloride Toxicity

**DOI:** 10.1155/2016/4154265

**Published:** 2016-02-29

**Authors:** Fernando G. Figueredo, Luciene F. Lima, Maria Flaviana B. Morais-Braga, Saulo R. Tintino, Pablo A. M. Farias, Edinardo F. F. Matias, José Galberto M. Costa, Irwin R. A. Menezes, Raimundo L. S. Pereira, Henrique D. M. Coutinho

**Affiliations:** Faculdade Leão Sampaio (FALS), 63180-000 Juazeiro do Norte, CE, Brazil

## Abstract

Mercury is a very dangerous metal when humans come into contact with it, whether through the air or skin or by ingestion. The aim of this work was to investigate the possible effects of the ethanol extract and fractions of* Lygodium venustum *Sw. against mercurium chloride toxicity towards* Escherichia coli* strain ATCC25922. The polyphenols and flavonoids present in the extract and fractions were quantified in mg equivalent of gallic acid/g sample and mg equivalent of quercetin/g sample, respectively. The* in vitro* FRAP method demonstrated the antioxidant activity of the samples. The antibacterial activity of the natural products was evaluated by microdilution method and by assays to elucidate the possible cytoprotective action when combining the natural products samples and mercurium chloride, utilizing the extract and fractions at a subinhibitory concentration. The results obtained in this work indicate that the ethanol extract and fractions of* L. venustum* are an alternative source of natural products with cytoprotective action, where this protection is correlated with antioxidant and chelating activity, due to the presence of total phenols and flavonoids.

## 1. Introduction

Heavy metals are elements naturally found in nature in small concentrations, but with the advent of industrialization and urbanization, the concentrations of these metals have been elevated to greater levels than natural ones, causing the contamination of aquatic and terrestrial ecosystems, and so they have consequently become one of the greatest environmental problems today [1]. At low concentrations, some metals are essential for growth of all types of organisms, from bacteria and plants to humans. Meanwhile, since they have bioaccumulative characteristics in the body, at high concentrations, they can become toxic by causing damage to biological systems [2].

An example of a heavy metal is mercurium chloride (HgCl_2_), which is very soluble in organic solvents and has high liposolubility when compared with the inorganic divalent form (Hg^+2^), which facilitates its permeability through biological membranes [3]. It can be dangerous if inhaled and extremely destructive to tissues of mucosal membranes and the upper respiratory tract. HgCl_2_ is toxic if absorbed through the skin, causing burns on the skin and in the eyes, and can be fatal if ingested, reaching mainly the kidneys, nerves, and gastrointestinal tract. In the aquatic environment, mercurium chloride is very toxic to organisms and can cause negative effects due to the bioaccumulation process [4].

Accordingly, some plants have mechanisms to chelate metals and metalloids in the rhizosphere, and they can be divided into two groups: plants that utilize efflux mechanisms, limiting absorption and/or transport to aerial parts, and those that use detoxification mechanisms based on internal immobilization or compartmentalization via the production of compounds that bind tightly to metals and metalloids [5].

Some studies have been conducted utilizing plants for phytoremediation of toxic metals, as can be seen in [6]. Francesconi et al. [7] and Oliveira et al. [8] demonstrated that* Pityrogramma calomelanos* and* Pteris vittata*, respectively, among other plants of the Cerrado, are capable of accumulating arsenic when present in the ground.

*L. venustum* (Lygodiaceae) is a fern with worldwide distribution and lianescent habit [9]. This species is used as a bioindicator of environmental degradation and in popular medicine among populations of South America [10].

The aim of this work was to evaluate the cytoprotective potential of* Lygodium venustum *Sw. (ethanolic extract and fractions) with respect to mercury chloride toxicity towards* Escherichia coli 25923*.

## 2. Materials and Methods

### 2.1. Plant Material

Leaves of* L. venustum *were collected in the city of Crato, Ceará, Brazil, in May of 2010 between 9:30 and 10:30 in the morning in a shaded area of Chapada do Araripe, at approximately 700 m altitude. The plant was identified by Dr. Antonio Álamo Feitosa Saraiva and voucher specimens were deposited at the Herbário Caririense Dárdano de Andrade-Lima of the Regional University of Cariri (URCA), under number #5569 HCDAL.

### 2.2. Preparation of Ethanol Extract (EELV) and Dichloromethane and Ethyl Acetate Fractions (DFLV and EAFLV) of* L. venustum*

Leaves were collected and 211.18 g was weighed, dried, and kept at room temperature. This material was powdered and extracted by maceration using 1 L of 95% ethanol solvent at room temperature. The mixture was allowed to stand for 72 h at room temperature. The extract was filtered and concentrated under vacuum in rotary evaporator at 60°C and 760 mmHg temperature and pressure, respectively [11, 12], obtaining 12.42 g of ethanol extract. Fractionation was performed using the ethanol extract, resulting in the fractions used in the tests (dichloromethane and ethyl acetate to yield 0.39 g and 0.52 g, resp.). The extract and fractions were diluted to 10 mg/mL of DMSO before the assays.

### 2.3. FRAP Assay

A modified method of Strain [13] was adopted for the FRAP assay. The stock solutions included 300 mM acetate buffer, pH 3.6, and 10 mM TPTZ (2,4,6-tripyridyl-S-triazine) solution in 40 mM HCl and 20 mM FeCl_3_·6H_2_O. The fresh working solution was prepared by mixing 25 mL acetate buffer, 2.5 mL TPTZ, and 2.5 mL FeCl_3_·6H_2_O. The temperature of the solution was raised to 37°C before using. Samples (0.15 mL) were allowed to react with 2.85 mL of FRAP solution for 30 min in the dark condition. Readings of the colored product (ferrous tripyridyltriazine complex) were taken at 593 nm. The standard curve was linear between 200 and 1000 *μ*M FeSO_4_. The results were expressed using mg equivalent of FeSO_4_/g of sample. Ascorbic acid was used as positive control [14].

### 2.4. Iron Chelating Activity

A modified method of Benzie and Strain [12, 13] was adopted for the assay. The principle is based on the formation of O-phenanthroline-Fe^2+^ complex and its disruption in the presence of chelating agents. The reaction mixture containing 1 mL of 0.05% O-phenanthroline in methanol, 2 mL of ferric chloride (200 *μ*M), and 2 mL of various concentrations ranging from 125 to 1000 *μ*g was incubated at room temperature for 10 min and the absorbance of the same was measured at 510 nm. The chelation activity content was extrapolated from a standard curve using FeSO_4_ (graded doses, 500–3000 *μ*g/mL) as a standard. The chelation activity was calculated as equivalents (mmol FeSO_4_/g extract).

### 2.5. Estimation of Total Phenol

Total phenolics were determined, in triplicate, by adding 200 *μ*L of each sample (100 to 800 *μ*g/mL of water) to 1 mL of Folin-Ciocalteau reagent (10%, v/v) followed by mixing for 1 minute. Next, 800 *μ*L of 7.5% sodium carbonate was added and the tubes were shaken for 30 seconds. After 1 hour, absorbance was read in a spectrophotometer at 765 nm. The blank contained all the reagents, but the extract was replaced with distilled water. The mean of three readings was used to determine the total phenolics, expressed as mg equivalent of gallic acid/g extract, interpolated from the calibration curve constructed with the gallic acid standards. The calibration curve for gallic acid was determined utilizing different concentrations of this compound (5 to 300 *μ*g/mL).

### 2.6. Estimation of Total Flavonoids

Solutions of extract and fractions were prepared (800 and 100 *μ*g/mL), and 1 mL of these was added to 1 mL 2% (w/v) aluminum chloride (AlCl_3_). The blank tube contained distilled water in place of AlCl_3_. After 30 minutes of incubation at ambient temperature, the absorbance was measured using a 415 nm filter. The assay was done in triplicate, and the mean was taken for determination of the quantity of total flavonoids and expressed as mg quercetin equivalents/g extract. The calibration curve for quercetin was determined utilizing different concentrations of this compound (0.78 to 200 *μ*g/mL) diluted in 80% ethanol.

### 2.7. Drugs

Gentamicin, amikacin, and neomycin were obtained from Sigma Chemical Corp., St. Louis, MO, USA. All of the drugs were dissolved in sterile water before use.

### 2.8. Culture Media

The following culture media were utilized in the assays:* heart infusion agar* (HIA) (Difco Laboratories Ltd.),* brain heart infusion *(BHI) (Acumedia Manufacturers Inc.), and M9 Tris.

### 2.9. Bacterial Material

The bacterial strain used was* E. coli* (EC-ATCC 25922). The strain was maintained on heart infusion agar (HIA, Difco Laboratories Ltd.). Before the test, the strains were grown for 18 h at 37°C in broth brain heart infusion (BHI, Difco Laboratories Ltd.).

### 2.10. Antibacterial Test (MIC) and Modulation of Mercuric Chloride Toxic Activity

MIC (Minimal Inhibitory Concentration) was determined in a microdilution assay [15, 16] utilizing an inoculum of 100 *μ*L of each strain, suspended in brain heart infusion (BHI) broth up to a final concentration of 10^5^ CFU/mL in 96-well microtiter plates, using twofold serial dilutions. Each well received 100 *μ*L of each extract solution. The final concentrations of the extracts varied in the range 512–8 *μ*g/mL. MICs were recorded as the lowest concentrations required to inhibit growth.

The minimal bactericidal concentration for the mercuric chloride was determined in M9 Tris by the microdilution assay utilizing suspensions of 10^5^ CFU/mL according to the McFarland scale and a drug concentration range of 500 to 0.24 *μ*M [15]. MBC was defined as the lowest concentration at which no growth was observed. For the evaluation of the extracts as modulators of resistance to the metal, MIC of the metal was determined in the presence or absence of extract (EELV) and fractions (DFLV and EAFLV) at subinhibitory concentrations (128 *μ*g/mL) and the plates were incubated for 24 h at 37°C. Reading replanting was performed using microdilution plates for these Petri dishes with heart infusion agar (HIA); they were incubated for 24 h at 37°C and thereafter results of MBC were observed. Each antibacterial assay for MBC determination was carried out in triplicate.

### 2.11. Statistical Analysis

Statistical analysis was performed using Prism*™* v4.0 software (GraphPad® Software, San Diego, California, USA). All chemical assays were carried out in triplicate and the data were expressed as means ± standard deviations (SD). One-way analysis of variance (ANOVA) for mean comparison and significant interhoney differences were calculated according to HSD Tukey's multiple-range test. The mean value for the minimum active dilution, in the antimicrobial activity test, was calculated from the triplicates. Linear regression plots were generated and correlations between antioxidant activities, FRAP, iron chelation, and total phenol and flavonoids contents were computed as Pearson's correlation coefficient (*r*) was used during this work to evaluate and correlate results between them. Differences at *p* > 0.05 were considered to be statistically significant.

## 3. Results

The contents of total phenols and flavonoids in the ethanolic extract of* L. venustum* and its fractions are presented in Table 1. The most representative concentration was that of fraction EAFLV for both types of compounds, which are plant secondary metabolites known for their antioxidant potential [14].

Table 2 gives the FRAP values for the ethanolic extract of* Lygodium venustum* and its respective fractions, demonstrating the reducing capacity in mg FeSO_4_/g sample. These data corroborate previously reported results [17] demonstrating that the extract and fractions of* Lygodium venustum* demonstrated an antioxidant activity.  Table 3 demonstrates the chelating capacity of EELV and its respective fractions.

Minimum inhibitory concentration (MIC) of the samples tested against the* E. coli *strain ATCC25922 showed comparatively the same result revealing MIC ≥ 1024 *μ*g/mL, demonstrating a low antibacterial activity [18]. A pilot study utilizing only DMSO was performed and showed no antibacterial or drug-modifying activity, indicating that the extract and fractions were not toxic to the strain assayed.

**Figure 1:**
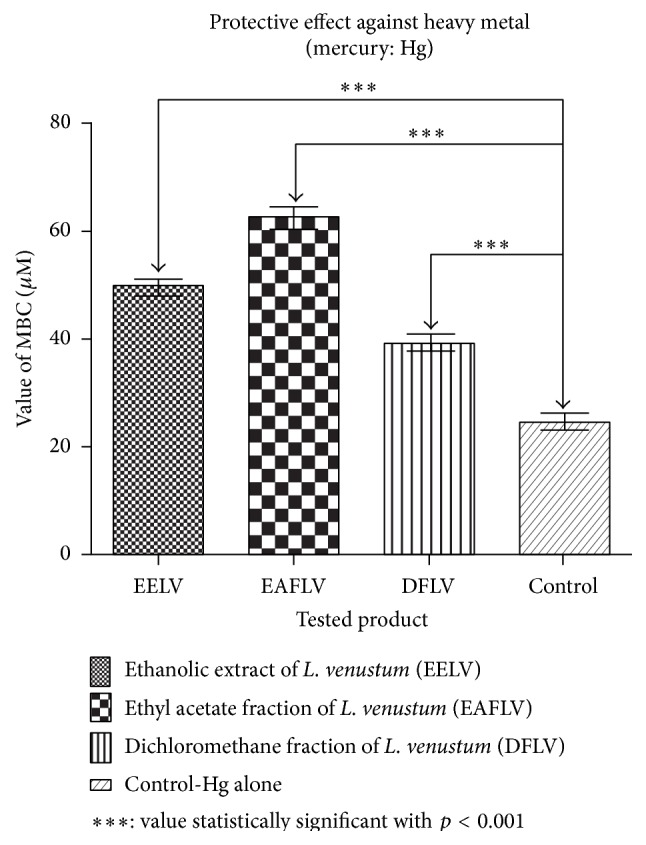
Chart showing the modulatory activity of the toxicity of mercury chloride towards* E. coli *25922 in the presence and absence of ethanol extracts of* Lygodium venustum* and its fractions and a concentration of MIC/8 (128 *μ*g/mL).

Figure 1 demonstrated the results of the evaluation of the effect of EELV and fractions against mercurium chloride toxicity. The results demonstrated that the extract and fractions reduced significantly the toxicity of mercurium chloride (*p* < 0.001) against* E. coli* 25922.

Table 4 demonstrates the correlation between the results obtained in this work, using the Pearson correlation coefficient (*r*). In analyzing the results, it was possible to infer that the minimum bactericidal concentration is related to antioxidant and chelating activity, due to the presence of total phenolics and flavonoids.

## 4. Discussion

Phytochemical prospecting reported by Morais-Braga et al. [19] revealed that the ethanol extract of* L. venustum* presents a variety of secondary metabolites, including phenols, phlorotannins, flavonols, flavonoids, xanthones, chalcones, flavonoids, and alkaloids, which showed the most diverse biological activities. The identification and quantification of phenolic compounds in EELV and its respective fractions were carried out by Morais-Braga et al. [20] using high-performance liquid chromatography (HPLC), which showed the presence of various compounds, including gallic acid, chlorogenic acid, caffeic acid, rutin, quercetin, and kaempferol, thereby demonstrating that the natural products studied contained flavonoids and phenolic acids and the derivates of the benzoic, phenylacrylic, and cinnamic acids. Chlorogenic and caffeic acids, as well as the flavonoid quercetin, were present in the extract as well as the fractions. Rutin, however, was not found in the dichloromethane fraction. Kaempferol was also absent in the ethanolic extract of this species. In the analysis of these data, quercetin was found in a large quantity in* L. venustum *due to the affinity for the solvent ethyl acetate [21] and this fraction presents a high concentration of this compound. Its occurrence is in agreement with that reported by the authors of [22], who affirmed that flavonoids are preferentially extracted by ethyl acetate.

The reducing capacity of a compound is an indicator of the antioxidant potential [14]. The results obtained demonstrated that EAFLV had the highest antioxidant activity, with equivalence of 68.43 mg FeSO_4_/g extract, demonstrating a capacity to reduce Fe^+3^ to Fe^+2^. This can be explained by the elution of phenolic and flavonoid compounds in this ethyl acetate, which can contribute directly to the antioxidant activity [17]. These results are in contrast to those obtained by [17], in which a greater antioxidant activity was found in the ethanol extract by the 2,2-diphenyl-1-picrylhydrazyl (DPPH) colorimetric assay. However, the difference between these results could be related to the different techniques used.

Iron is a divalent cation essential for life, being necessary to the oxygen transport, respiration, and the enzymatic activity. However, it is an extremely reactive metal and catalyzes oxidative damage in lipids, proteins, and other cellular components [13].

It is evident by this study that the natural products have chelating activity, binding and reducing Fe^+3^ to Fe^+2^. According to Duh et al. [23], this activity can be due to the presence of polyphenols, which prevent the cellular damage caused by the free radicals formed by the reduction of these ions [23].

The chemical form of mercury and the type of exposure affect its distribution in tissues and toxicity. The principal targets of exposure to mercury chloride are the kidneys, liver, blood, intestinal epithelium, and lungs [24, 25]. Inside the cells, mercury can interact with several biomolecules, such as glutathione and sulfhydryl groups of proteins present in antioxidants, DNA repair enzymes, and proteins involved in homeostasis, altering their normal activity [24].

A combination of some natural products rich in phenolic compounds with mercury chloride can be an alternative for minimizing the contamination with this metal, since their combination can cause an antagonistic effect, reducing the toxicity of mercurium chloride, increasing the necessary dose to cause toxic damage against the cells.

## 5. Conclusion

The results obtained in this work indicated that the ethanol extract and fractions of* Lygodium venustum* are an alternative source of natural products with cytoprotective activity. These results demonstrated that the extract and fractions had an antagonistic effect against mercurium chloride toxicity in a bacterial model using the strain* E. coli* 25922 and that this antagonism was correlated with the antioxidant and chelating activity, possibly due to the presence of phenolic acids and flavonoids.

## Figures and Tables

**Table 1:** The content of total phenol and flavonoids of the ethanolic extract and fractions of *L. venustum*.

Number	Samples	Content of total phenols (mg gallic acid equivalent/g of sample) (±SEM)	Content of flavonoids (mg equivalent of quercetin/g of sample) (±SEM)
(1)	EELV	37.10 ± 0.08	16.29 ± 0.02
(2)	DFLV	17.77 ± 0.07	10.80 ± 0.09
(3)	EAFLV	68.43 ± 0.04	40.20 ± 0.04

EELV: ethanol extract of *Lygodium venustum*; FDLV: dichloromethane fraction of *Lygodium venustum*; EAFLV: ethyl acetate fraction of *Lygodium venustum*.^*∗*^All values are expressed as mean ± SEM for three determinations.

**Table 2:** FRAP test of the ethanolic extract and fractions of *L. venustum*.

Number	Samples	Reductive activity (mg equivalent of FeSO_4_/g of sample) (±SEM)
(1)	EELV	37.1 ± 0.04
(2)	DFLV	17.77 ± 0.02
(3)	EAFLV	68.43 ± 0.03

^*∗*^All values are expressed as mean ± SEM for three determinations.

**Table 3:** Chelating activity of iron of the ethanolic extract and fractions of *L. venustum.*

Number	Samples	Chelating activity (mg equivalent of FeSO_4_/g of sample) (±SEM)
(1)	EELV	42.64 ± 4.08
(2)	DFLV	23.65 ± 1.40
(3)	EAFLV	59.24 ± 1.45

^*∗*^All values are expressed as mean ± SEM for three determinations.

**Table 4:** Correlation between the results obtained in this work.

	MBC	AA	CA	Flavonoids	Phenols
MBC		0.997	0.990	0.965	0.997
AA	0.997		0.999	0.944	0.990
CA	0.993	0.999		0.928	0.982
Flavonoids	0.965	0.944	0.928		0.982
Phenols	0.997	0.990	0.982	0.982	

MBC: minimum bactericidal concentration; AA: antioxidant activity; CA: chelating activity.
